# Medical Students and Graduates in the United States during the Session of 1858–59

**Published:** 1859-05

**Authors:** 


MEDICAL STUDENTS AND GRADUATES IN THE UNITED STATES DURING THE SESSION OF 1858–59.—So far as we have been able to learn, the subjoined list of the medical classes in the different schools is correct. All the institutions of this country are not represented, as we have not been able to gather authentic facts in relation to them. By comparing this list with that published a year ago in this journal, it will be seen that more students were in attendance at the different colleges than the year previously.

| | Students. | Graduates. |
|---|---|---|
| Jefferson Medical College, | 570 | 256 |
| University of Pennsylvania, | 409 | 144 |
| Pennsylvania Medical College, | 109 | 33 |
| Philadelphia College of Medicine, | 75 | 18 |
| University of New York, | 351 | 128 |
| College of Physicians and Surgeons, | 180 | 58 |
| New York Medical College, | 107 | 25 |
| University of Louisiana, | 335 | 98 |
| New Orleans School of Medicine, | 140 | 36 |
| University of Nashville, | 436 | 103 |
| Shelby Medical College, | 53 | 11 |
| St. Louis Medical College, | 135 | 40 |
| Missouri Medical College, | — | 23 |
| Kentucky School of Medicine, | 103 | 28 |
| University of Louisville, | — | 35 |
| University of Michigan, | 143 | — |
| University of Buffalo, | 67 | 13 |
| Medical College of Georgia, | 165 | 58 |
| Atlanta Medical College, | 136 | 39 |
| Oglethorpe Medical College, | — | 13 |
| Savannah Medical College, | 34 | 8 |
| Rush Medical College, | — | 31 |
| Transylvania Medical College, | 18 | 6 |
| Harvard University, | 139 | 30 |
| Medical College of Ohio, | 139 | 32 |
| Medical College of South Carolina, | 195 | — |
| Virginia Medical College, | 70 | 20 |
| University of Virginia, | 107 | — |
| Medical School of Maine, | 50 | — |
| University of Vermont, | 80 | 20 |
| Albany Medical College, | — | 48 |
| Dartmouth Medical College, | — | 9 |

